# Gualou Xiebai Banxia decoction ameliorates Poloxamer 407-induced hyperlipidemia

**DOI:** 10.1042/BSR20204216

**Published:** 2021-06-14

**Authors:** Mingzhu Luo, Rong Fan, Xiaoming Wang, Junyu Lu, Ping Li, Wenbin Chu, Yonghe Hu, Xuewei Chen

**Affiliations:** 1 Affiliated Hospital of Chengdu University of Traditional Chinese Medicine, Chengdu 611137, Sichuan, China; 2 Department of Operational Medicinal Research, Tianjin Institute of Environmental and Operational Medicine, Tianjin 300050, China; 3 Central Laboratory, Tianjin Xiqing Hospital, Tianjin 300380, China; 4 Department of Pharmacology Research, Southwest Medical University, Luzhou 646000, Sichuan, China; 5 Department of Traditional Chinese Medicine, The General Hospital of Western Theater Command, Chengdu 611137, Sichuan, China

**Keywords:** GLXBBX, liver steatosis, LPL, P407

## Abstract

**Ethnopharmacological relevance:** Gualou Xiebai Banxia (GLXBBX) decoction is a well-known traditional Chinese herbal formula that was first discussed in the *Synopsis of the Golden Chamber* by Zhang Zhongjing in the Eastern Han Dynasty. In traditional Chinese medicine, GLXBBX is commonly prescribed to treat cardiovascular diseases, such as coronary heart disease and atherosclerosis.

**Objective:** The present study aimed to examine GLXBBX’s preventative capacity and elucidate the potential molecular mechanism of Poloxamer 407 (P407)-induced hyperlipidemia in rats.

**Materials and methods:** Both the control and model groups received pure water, and the test group also received a GLXBBX decoction. For each administration, 3 ml of the solution was administered orally. To establish hyperlipidemia, a solution mixed with 0.25 g/kg P407 dissolved in 0.9% normal saline was injected slowly into the abdominal cavity. At the end of the study, the rats’ plasma lipid levels were calculated using an automatic biochemical analyzer to evaluate the preventative capability of the GLXBBX decoction, and the serum and liver of the rats were collected.

**Results:** The GLXBBX decoction significantly improved P407-induced hyperlipidemia, including increased plasma triglycerides (TGs), aspartate aminotransferase (AST) elevation, and lipid accumulation. Moreover, GLXBBX decoction treatment increased lipoprotein lipase (LPL) activity and mRNA expression of LPL. Furthermore, GLXBBX significantly suppressed the mRNA expression of stearoyl-CoA desaturase (SCD1).

**Conclusion:** GLXBBX significantly improved P407-induced hyperlipidemia, which may have been related to enhanced LPL activity, increased LPL mRNA expression, and decreased mRNA expression of SCD1.

## Introduction

Abnormal blood lipid metabolism is very common and a major risk factor for cardiovascular disease, though it is also involved in the development of a variety of diseases [1]. Hyperlipidemia involves abnormal blood lipid metabolism, which includes elevated levels of serum triglycerides (TGs), total cholesterol (TC), low-density lipoprotein (LDL), cholesterol, and free fatty acids and decreased levels of serum high-density lipoprotein cholesterol [2]. According to epidemiological investigations, both elevated plasma TG and TC are emerging as independent risk factors for metabolic syndrome, type 2 diabetes, hepatic steatosis, and atherosclerotic cardiovascular disease [3,4]. Hypertriglyceridemia is generated by a complex interplay among several genetic susceptibilities and environmental factors that affect the production and clearance of TG-rich lipoproteins. Among these factors, the etiopathogenesis of hyperlipidemia consists of the dysregulation of gene expression during the synthesis and decomposition of TGs. Plasma TG levels increase as a result of both the absorption of dietary fat by intestinal cells and the restricted catabolism of TGs. The catabolism of TGs is mainly regulated by plasma lipoprotein lipase (LPL), which gradually hydrolyzes TGs into free fatty acids and glycerol, which are released into the bloodstream for tissue oxidation [5]. While adipocytes and myocytes are the main source of LPL, the vast majority of LPL activity occurs in capillary endothelial cells. A partial or complete LPL deficiency results in an inability to hydrolyze TGs in chylomicrons and very-LDLs, causing mild to severe hypertriglyceridemia [6,7]. Poloxamer 407 (P407) has been shown to induce massive hyperlipidemia by directly inhibiting the heparin-releasable fraction of LPL [8,9].

Hyperlipidemia, which makes the liver more susceptible to damage, is the first step in hepatic lipid accumulation. The synthesis of hepatic TGs is mainly regulated by the combined action of fatty acid synthase (FAS), acetyl-CoA carboxylase (ACC), and stearoyl-CoA desaturase (SCD1) [10–12]. In addition, inflammatory cytokines, oxidative stress, and anti- and pro-inflammatory molecules play important roles in lipid metabolic disorders.

Effective lipid-lowering therapy is essential for hyperlipidemia. Mild hyperlipidemia is usually treated through the introduction of lifestyle modifications, and pharmacotherapy is indicated for more severe cases of hyperlipidemia. Traditional Chinese medicine is a classic and effective therapeutic approach that is both diverse and unique. Gualou Xiebai Banxia (GLXBBX) decoction was first discussed in the *Synopsis of the Golden Chamber* written by Zhang Zhongjing. It mainly treats phlegm and stasis obstructing chest discomfort syndrome. Herbal components of GLXBBX have been identified as Trichosanthes, *Allium macrostemon*, Pinellia ternata, and rice wine. GLXBBX is effective for cardiovascular diseases and is widely used during clinical surgery in China to treat coronary heart disease and atherosclerosis and has a good curative effect [13–15]. GLXBBX appears to have beneficial effects on the electrocardiogram and a reduction in angina symptoms in participants with angina pectoris [16]. The Dan-Lou prescription, which is a transformation of the GLXBBX decoction, inhibits foam cell formation induced by ox-LDL via the TLR4/NF-κB and PPARγ signaling pathways [17]. Although the lipid-lowering effect of GLXBBX has previously been proposed, the molecular mechanism has not been clarified.

In the present study, we attempted to depict the underlying mechanism by which GLXBBX improves hyperlipidemia induced by P407 based on the interaction between lipid-lowering and LPL activity. After verification, we found that GLXBBX alleviated hyperlipidemia induced by P407 by activating LPL activity, and GLXBBX inhibited early hepatic steatosis by antagonizing the mRNA overexpression of SCD1, blocking hepatic TG synthesis. In conclusion, we provided evidence that GLXBBX treatment effectively attenuates hyperlipidemia by activating LPL activity.

## Materials and methods

### Reagents and chemicals

P407 and d-glucose were purchased from Beijing Solarbio Technology Co., Ltd. (Beijing, China). TRNzol Universal total RNA extraction reagent and RNase/DNase-free ddH_2_O were purchased from Tiangen Biochemical Technology Co., Ltd. (Beijing, China). TB Green Premix Ex Taq II was purchased from Takara Biomedical Technology Co., Ltd. and Trans DNA Marker II and DNase I were purchased from TransGen Biotech (Beijing, China). The Hematoxylin and Eosin (H&E) staining solution was purchased from Nanjing Jiancheng Bioengineering Institute. The Oil Red O staining solution was purchased from Servicebio Co., Ltd. (Wuhan, China).

### Composition and preparation of the GLXBBX decoction

The composition of GLXBBX decoction is listed in Table 1. GLXBBX herbal slices (1320 g) were obtained from Anguo Shenhao Pharmaceutical Co., Ltd., Baoding, China and the proportions of the components in the GLXBBX formula were maintained. Rice wine was purchased from Shaoxing Nuerhong Brewing Co., Ltd., and the content was 15.5% alcohol/volume. The herbal mixture was crushed and soaked with 3500 ml of rice wine overnight, and pure water was added to the medicinal herbs, which were decocted on a slow fire, and the extract was filtered twice. The resulting solution was concentrated to obtain exactly 1320 ml, which was cooled to room temperature and stored at −20°C after cooling for later use. The extract was rewarmed to 37°C before use.

**Table 1:** The composition of GLXBBX decoction

Pharmaceutical	Pīnyīn	Composition
*Trichosanthes*	*Guā*lóu	37.50%
*Allium macrostemon*	*Xiè*baí	25%
*Pinellia ternata*	*Bà*nxià	37.50%
Rice wine	*Huá*ngjiǔ	15.5% vol

### Animals

Male Wistar rats (6–8 weeks old) were purchased from Beijing Weitong Lihua Animal Laboratory Technology Co., Ltd. (Beijing, China). They were housed in an animal room at a consistent temperature (23 ± 2°C) and humidity and maintained on a 12-h light and dark cycle. Rats were divided randomly into two groups: one group received the GLXBBX decoction (5 g/kg) (GLXBBX group; *n*=11) and the other group (*n*=14) received an equal volume of pure water. One week later, the pure water group was randomized into two groups: the P407 group (*n*=8) and the control group (*n*=6). Both the P407 group and the GLXBBX decoction group were given bolus intraperitoneal (i.p.) injections of P407 (0.5 g/kg) to establish the hyperlipidemia model [18], and the control group received 0.9% saline (*n*=6), twice a week for 4 weeks. P407 was dissolved in cold saline (0.9%) and kept overnight at 4°C for complete dissolution according to the cold method.

All rats were killed on the last day at the same time. After anesthesia with sodium pentobarbital (40 mg/kg, i.p.), blood was drawn from the abdominal aorta, anticoagulated using heparin sodium, and plasma was separated centrifugally. All experimental protocols were approved by the Animal Ethics Committee of the Institute of Environmental Medicine and Occupational Medicine, Institute of Military Medicine, Academy of Military Sciences (IAUC No. AMMS-04-2020-053).

### Measurement of plasma lipid profiles, liver tissue lipid content, and the liver/body ratio

The intact liver was removed after the rats were sacrificed, and the liver/body weight ratio was calculated to evaluate the amount of fat deposited in the liver. The TG and TC levels in the plasma were measured using a fully automatic bio-analysis machine (Mindray BS 180, China). TG and TC levels in liver tissue homogenates were determined using an ELISA kit (Nanjing Jiancheng, China).

### Oral glucose tolerance tests

Rats were fasted overnight for 12–14 h with free access to water, and glucose was administered orally at 2.5 g/kg of body weight. Blood glucose was measured from the tail using an automatic glucometer (OneTouch UltraEasy, U.S.A.) at baseline (0), 15, 30, and 120 min after administration.

### Analysis of serum glucose, alanine aminotransferase, and aspartate aminotransferase

Plasma glucose, alanine aminotransferase (ALT), and aspartate aminotransferase (AST) levels were measured using a biochemical analyzer (Mindray BS 180, China), and the AST/ALT ratio was calculated.

### Plasma LPL activity

LPL activity was determined using an LPL activity kit (Mrybio, China) according to the manufacturer’s instructions.

### Histopathological observation

Liver tissue was fixed in 4% paraformaldehyde. After 2 days, the tissue was dehydrated using ethanol concentrations (from 70 to 100%) and subsequently embedded in paraffin. The tissue was sliced into 5-μm-sections and dried overnight for staining. The tissue sections were then stained with H&E and Toluidine Blue after deparaffination and rehydration. Each section was dehydrated with ethanol, cleared with xylene, and mounted on slides using a mounting medium. Lipid deposition in liver cells was assessed by Oil Red O staining. The liver tissue sections were stained with 0.5% Oil Red O solution for 1 h and then differentiated in a 60% propylene glycol solution for 5 min. The slides were observed and measured in five high-power fields using a light microscope (Leica, Germany) at 40× magnification. At least five sections of four mice from each group were randomly selected and analyzed.

### Quantitative real-time polymerase chain reaction

Liver tissue was obtained, flash frozen, and stored at −80°C until processing. Total RNA was isolated using the TRIzol reagent. RNA was reverse transcribed into cDNA using the RevertAid First Strand cDNA Synthesis Kit (Thermo Fisher, U.S.A.). The mRNA expression levels of *FAS*, *ACC*, and *SCD1* were evaluated by quantitative reverse transcription polymerase chain reaction (qRT-PCR) analysis using the StepOne Real-Time PCR System (ABI, U.S.A.). The primer sequences are listed in Table 2. Target mRNA expression in each sample was normalized to that of the housekeeping gene,* GAPDH*. The 2^−ΔΔ*C*_t_^ method was used to calculate relative mRNA expression levels.

**Table 2:** Primer sequences used in the present study

Primer name	Primer sequence (5′–3′)
LPL	Forward: AGCCCCTAGTCGCCTTTCTCCTG
	Reverse: GCCTTGCTGGGGTTTTCTTCATTC
Fas	Forward: TTGATGAAGAGGGACCATAAAGA
	Reverse: TGGGAACAAGGCATTAGGGT
Acc	Forward: CCGTGAAGGGCTACCTCTAATG
	Reverse: ATCACAACCCAAGAACCACCC
Scd1	Forward: CCTCATCATTGCCAACACCAT
	Reverse: GCACAAGCAGCCAACCCAC
Gapdh	Forward: TGCCACTCAGAAGACTGTGG
	Reverse: TTCAGCTCTGGGATGACCTT

### Western blotting assay

Whole liver tissue lysates were generated using urea lysis buffer and run on 10% SDS/PAGE gel. Proteins were transferred on to PVDF membranes (Millipore, U.S.A.), and incubated with antibodies against FAS (1:1000, Abcam), ACC (1:1000, Abcam), SCD1 (1:1000, Abcam), respectively, followed by incubation with corresponding secondary antibodies conjugated to horseradish peroxidase. The β-Actin (1:1000, Beyotime Biotechnology) Western blotting was performed for each membrane as a loading control. Immunoreactive proteins were visualized using ECL reagents (Millipore, U.S.A.).

### Statistical analysis

All results are presented as the mean ± SD. Multigroup comparisons were performed using one-way analysis of variance followed by Dunnett’s test. GraphPad Prism software was used to perform the statistical analysis (version 7.0; GraphPad Software, Inc., San Diego, CA, U.S.A.). Differences were considered statistically significant at *P*<0.05.

## Results

### GLXBBX treatment ameliorates hyperlipidemia induced by P407

**Figure 1:**
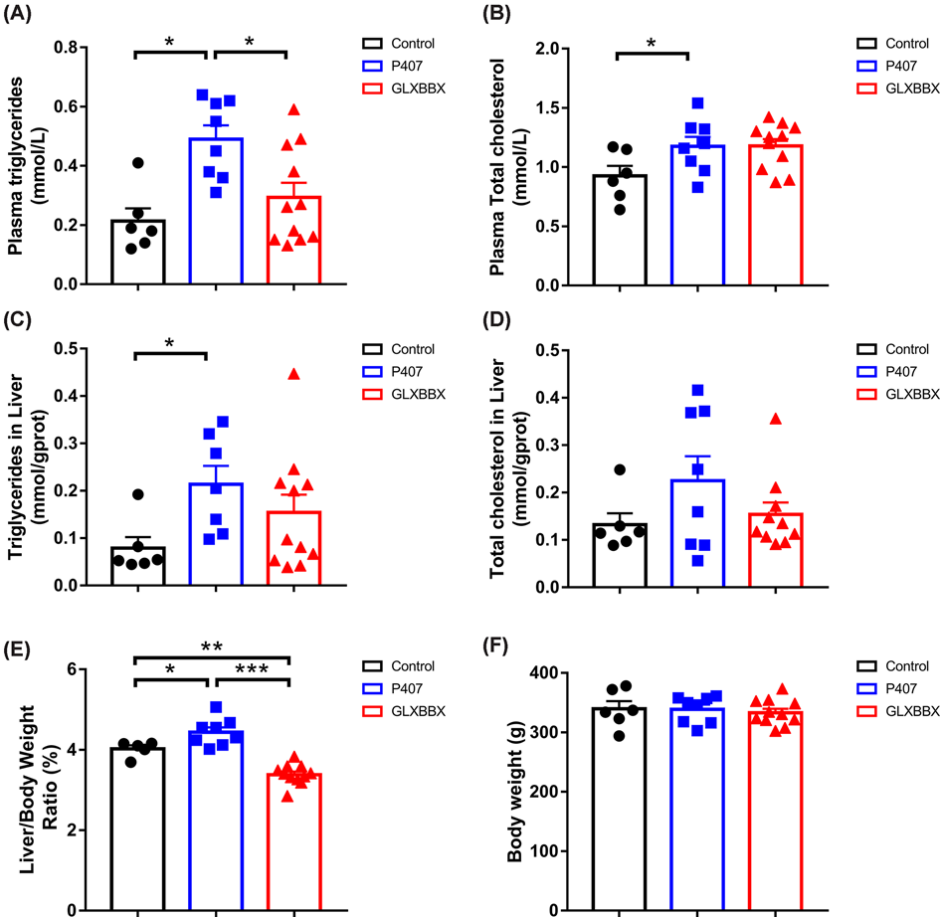
GLXBBX decoction modulates plasma and hepatic-rich lipid levels induced by P407 (**A**,**B**) Plasma TGs (A) and TC (B). (**C**,**D**) Hepatic TGs (C) and TC (D). (**E**) Liver wet weigh to carcass body weight ratio in the control, model, and GLXBBX-treated rats. (**F**) Carcass body weights for the different groups. *P<0.05; **P<0.01; ***P<0.001.

As previously described, P407, a highly efficient inhibitor of LPL, has been extensively used to induce hyperlipidemia in rodents and other experimental animals [19,20]. To investigate the potential role of GLXBBX decoction on lipid lowering, we first estimated its phenotype in the plasma and liver of rats. The carcass weight was similar in all groups (*P*>0.05). Both the plasma TG and TC levels were clearly higher in the group that was treated with P407 compared with their control littermates (Figure 1A,B). Furthermore, an over-accumulation of TG was observed in the liver of the P407 group (Figure 1C), with a significant elevation in the liver/body weight ratio (*P*<0.05) (Figure 1E) and a tendency toward elevated TC levels (Figure 1D).

After treatment with GLXBBX decoction, rats showed a 24% reduction in the liver/body weight ratio (*P*<0.05) (Figure 1E) and a 40% reduction in the plasma TG level (*P*<0.05) (Figure 1A). Both TG and TC levels in the livers of the GLXBBX-treated rats were 28 and 32% lower, respectively, while there were no obvious changes in TG and TC levels in the liver tissue.

### GLXBBX decoction provides protection against hepatotoxicity induced by P407

**Figure 2:**
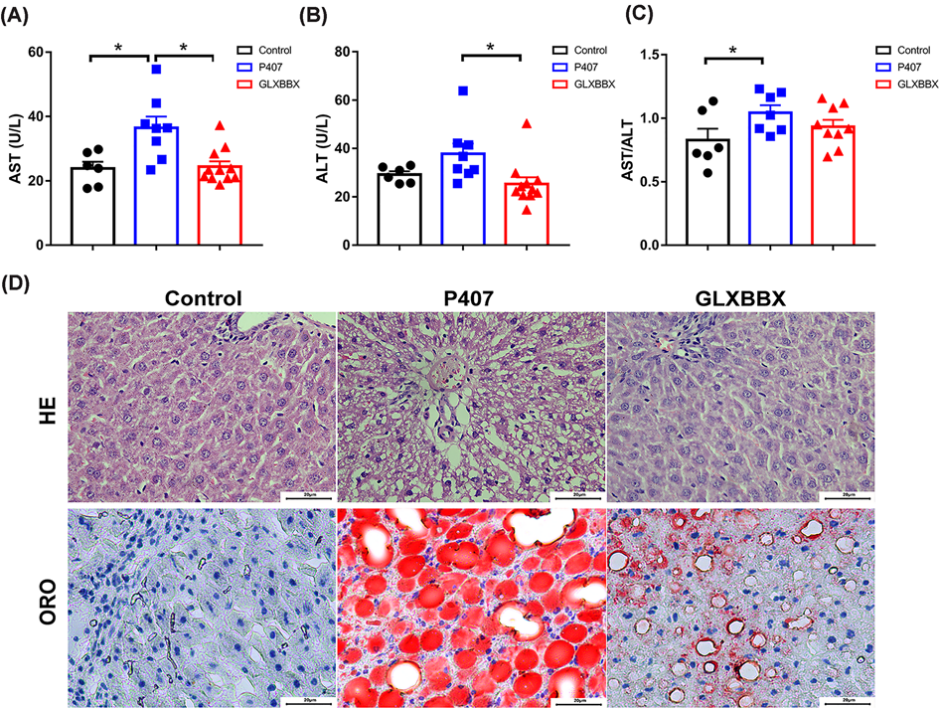
GLXBBX decoction improves hepatic function and reduces hepatic lipid accumulation (**A**,**B**) Plasma liver enzymes, AST (A) and ALT (B). (**C**) AST/ALT ratios for the different groups. (**D**) H&E staining of hepatic tissue (D, top) and Oil Red O staining of hepatic tissue (D, bottom). *P<0.05.

The liver plays an important role in lipid metabolism. The initial step in hepatic metabolism of lipoproteins is their transfer through 100–200 nm pores (fenestrations) in the liver sinusoidal endothelial cells prior to receptor-mediated uptake [21]. H&E staining of the liver showed that the control group had normal hepatic lobules, hepatic cords orderly arranged around the central vein, and clearly visible hepatic sinuses (Figure 2D upper). Conversely, normal hepatocytes were almost invisible in the liver tissue of the P407-treated rats, with enlarged hepatocytes, disordered hepatic cords, numerous fat vacuoles, and lightly stained cytoplasm. Oil Red O staining showed that there was an accumulation of lipid droplets in the P407 group, and GLXBBX treatment significantly decreased P407-induced hepatic fat deposition (Figure 2D bottom).

For the GLXBBX-treated rats, hepatic lipid accumulation was significantly reduced. To further understand the effect of P407 on the liver and the GLXBBX decoction-associated lipid-lowering expression pattern, we assessed liver function tests. The increased serum concentrations of AST and ALT in those treated with P407 were down-regulated by GLXBBX. Compared with the P407-treated rats, the AST and ALT levels in the GLXBBX-treated rats decreased sharply by 32 and 33%, respectively (*P*<0.05) (Figure 2A,B). The AST/ALT ratio, which is considered an alternative marker of hepatic steatosis, was significantly increased with P407 (*P*<0.05) (Figure 2C).

### P407-induced impaired glucose tolerance and high random blood glucose levels

**Figure 3:**
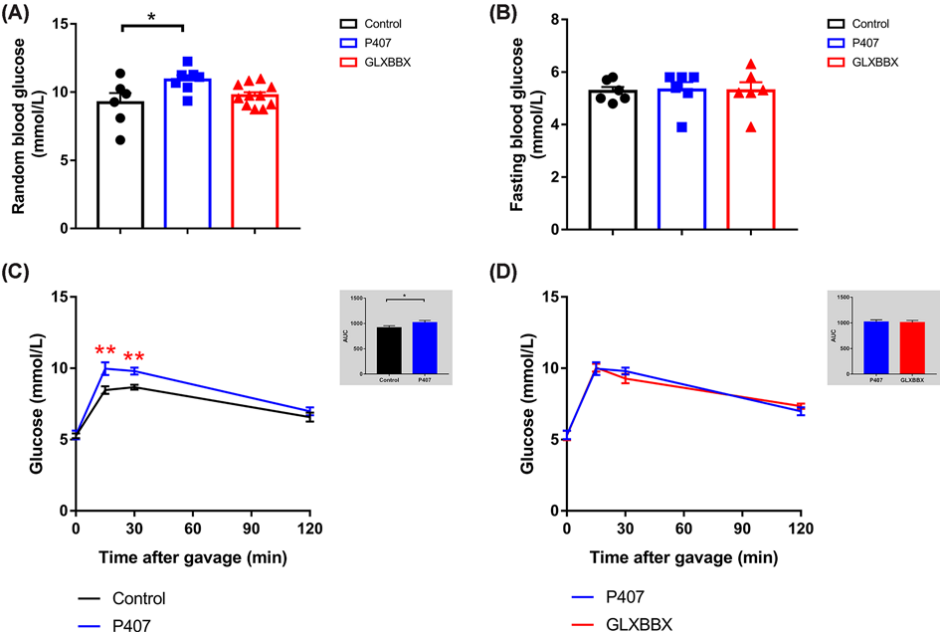
P407-induced hyperlipidemia accompanied by high random blood glucose levels and impaired glucose tolerance (**A**) Random blood glucose levels for the different groups. (**B**) Fasting blood glucose levels for the different groups. (**C**,**D**) Time course of blood glucose levels after 2.5 g/kg glucose gavage and area under the curve (AUC) in the control and P407 groups (C) and the P407 and GLXBBX groups (D) are shown. *P<0.05; **P<0.01.

Although fasting blood glucose levels were normal in all treatment groups, the model rats with P407-induced hyperlipidemia had more impaired glucose tolerance compared with the controls. The model rats’ ability to process glucose was reduced, and the blood glucose levels at 15 and 30 min were significantly higher in the model rats compared with the controls (*P*<0.01) (Figure 3), though there were no obvious differences between the model rats and the GLXBBX-treated rats. In addition, random blood glucose levels were increased in the P407 group (*P*<0.05) (Figure 3A). Thus, overt impaired glucose tolerance was observed in rats with P407-induced hyperlipidemia.

### GLXBBX decreases the expression of SCD1

**Figure 4:**
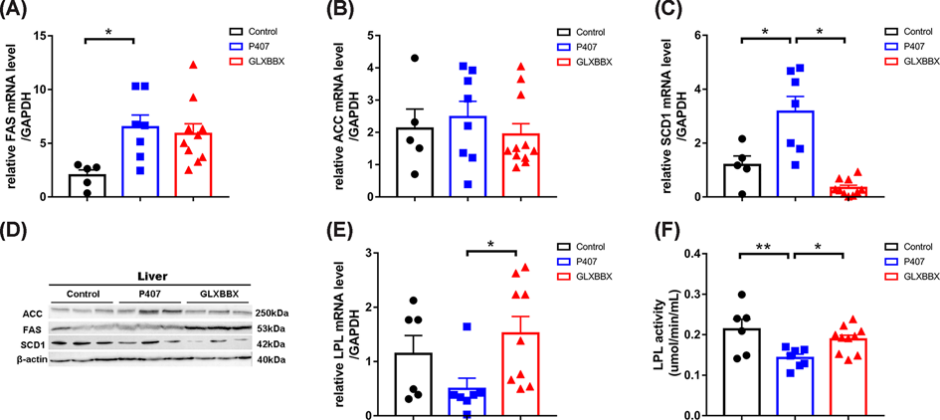
Expression of key enzymes related to TG synthesis and decomposition modulated by GLXBBX decoction (**A**–**C**) The mRNA levels of TG synthesis in hepatic tissue of the control, P407, and GLXBBX-treated rats. (**D**) The protein levels of TG synthesis in hepatic tissue of the control, P407, and GLXBBX-treated rats. (**E**) The mRNA levels of LPL in brown adipose tissue of the control, P407, and GLXBBX-treated rats. (**F**) Plasma LPL activity of the different groups are shown. *P<0.05; **P<0.01.

Sterol regulatory element binding protein (SREBP1c), a key transcription factor for lipid synthesis, activates downstream target genes such as *FAS*, *ACC*, *SCD1*, and other key enzymes involved in lipid synthesis, which promote TG synthesis and lipid deposition [22]. The expression of FAS, ACC, and SCD1 is dysregulated in the livers of rats with fatty liver and obesity [23]. Therefore, the mRNA expression of these key enzymes was investigated using RT-qPCR, and the results are shown in Figure 4 and Supplementary Data. The mRNA expression of FAS, ACC, and SCD1 in the model rats increased to varying degrees, though the expression of FAS and SCD1 in the model rats significantly differed from that of the controls.

SCD1 is involved in the final step of TG synthesis in the liver. The mRNA expression of SCD1 in GLXBBX-treated rats was significantly lower than that in the P407 group (*P*<0.05) (Figure 4C). Furthermore, Western blot also confirmed that the protein expression level of SCD1 in the GLXBBX group was remarkably decreased than that in the P407 group (Figure 4D).

### GLXBBX decoction enhances LPL activity

LPL is a regulatory enzyme involved in the hydrolysis of TGs in both chylomicrons and very LDL particles and in the conversion of high-density lipoproteins. The mRNA expression level of LPL of rats in the P407 group decreased compared with control group, while the treatment of GLXBBX could restore the drop in LPL expression level caused by P407 (Figure 4E). The LPL activity in the plasma of the P407-treated rats sharply decreased 33% (*P*<0.01) compared with the controls. Furthermore, the LPL activity in the GLXBBX-treated rats was considerably higher than those in the P407 group (*P*<0.05) (Figure 4F and Table 3).

**Table 3:** Comparison of plasma LPL activity in different groups 
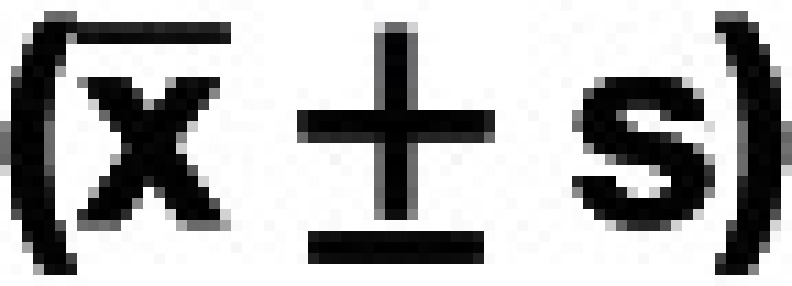

	Plasma LPL activity
Control group	0.2 ± 0.06
P407 group	0.14 ± 0.02^1^
GLXBBX group	0.19 ± 0.03^2^

Compared with control group.

1 *P*<0.01; compared with P407 group.

2 *P*<0.05.

## Discussion

LPL deficiency has been reported to contribute to hypertriglyceridemia in previous studies [6], and the rat model induced by P407 showed decreased LPL expression and activity and increased plasma TG levels [8]. This study explored whether the oral administration of GLXBBX could improve hyperlipidemia induced by the i.p. injection of P407, thereby protecting cardiovascular function. Previous studies of GLXBBX have established a basis for the clinical application of GLXBBX in cardiovascular diseases accompanied by hyperlipidemia, which has promoted further investigation into the efficacy of GLXBBX in the treatment of hyperlipidemia.

The liver plays an important role in lipid metabolism. The initial step in the hepatic metabolism of lipoproteins is their transfer through 100–200 nm pores (fenestrations) in the liver sinusoidal endothelial cells prior to receptor-mediated uptake [21]. P407 is a ubiquitous manmade surfactant and non-ionic detergent composed of repeating polyoxyethylene and polyoxypropylene units and is chemically a polyether-based compound [24]. P407 is taken up by the liver, resulting in dramatic defenestration of liver sinusoidal endothelial cells [25]. It has been suggested that the mechanism of P407-induced hypertriglyceridemia involves elevated TG levels resulting from the inhibition of LPL [20], and P407-induced hypercholesterolemia involves the indirect stimulation of 3-hydroxy-3-methylglutaryl coenzyme A reductase and decreased LDL receptor expression in the liver [26,27]. Dramatic fenestration has been caused by P407 in isolated liver sinusoidal endothelial cells. Based on this model, we found that hyperlipidemia was accompanied by liver steatosis and abnormal glucose metabolism. We demonstrated that GLXBBX treatment inhibits hyperlipidemia and ameliorates P407-induced hepatic damage, and we further investigated the mechanism by which GLXBBX could improve hypertriglyceridemia. As shown in the present study, the clinical condition of rats can be improved by GLXBBX through a significant reduction in plasma TG levels, though with no resulting change in plasma TC. Gualou Xiebai decoction is a classic prescription in Chinese medicine used for the treatment of cardiac conditions, but also serves many other functions, including antioxidation, anti-apoptosis, and anti-inflammation [28]. Decoctions containing Banxia have also been used for hyperlipidemia treatment. The Banxia Baizhu Tianma decoction is made up of six Chinese herbal medicines, including Banxia. Research on rats has shown that the Banxia Baizhu Tianma decoction can reduce serum TC, TG, LDL cholesterol, apolipoprotein B, superoxide dismutase, and malondialdehyde in rats with hyperlipidemia [29]. Moreover, Jiang et al. found that the Banxia Baizhu Tianma decoction has a significant effect on body weight and TC in mice with obesity-related hypertension, reducing lipid deposition in the roots of arteries, protecting endothelial cells, reducing apoptosis, decreasing the migration rate caused by oxidized LDLs, and maintaining cell morphology [30].

The metabolic pathways and primary metabolic sites of TGs are different from those of TC metabolism. LPL, an enzyme that is secreted by adipocytes and myocytes, acts as an intravascular TG hydrolase by converting TGs into fatty acids for utilization by vital tissues once it reaches its site of action in the capillary lumen. To investigate the efficacy of GLXBBX from a histological perspective, macroscopic and microscopic scores were obtained. GLXBBX was found to have a significant intervention effect, through the reduction in tissue damage and improvement in liver steatosis. GLXBBX decoction has been found to down-regulate TG levels by increasing LPL activity. Previous studies [5] have established that glycosylphosphatidyl inositol-anchored high-density lipoprotein-binding protein 1 (GPIHBP1) is needed for the transportation of LPL into the capillaries, lipoprotein margination, and the preservation of LPL structure and activity. Deficiency in either LPL or GPIHBP1 impairs TG hydrolysis, resulting in severe hypertriglyceridemia. LPL activity in the tissues is regulated by angiopoietin-like proteins [31]. Angiopoietin-like proteins inactivate LPL by converting LPL homodimers into monomers, rendering them highly susceptible to spontaneous unfolding and the loss of enzymatic activity. Future research is needed that focuses on the properties of LPL and explores the mechanism by which GLXBBX regulates LPL activity. GPIHBP1 and angiopoietin-like proteins are good potential points of penetration.

Although there was a trend of hepatic lipid down-regulation in the GLXBBX decoction group, this finding was not statistically significant, which might have been due to the small sample size. The antihyperlipidemic effects of the GLXBBX decoction were confirmed by analyzing the mRNA expression of lipogenesis-related genes, revealing that GLXBBX reduced the mRNA expression of SCD1 and FAS. Therefore, the antihyperlipidemic effects of GLXBBX could be attributed to the suppression of these lipogenic genes. Interestingly, it was reported that *Ganoderma lucidum* exhibited anti-obesity activity by down-regulating FAS, SCD1, and SREBP1c, and also displayed antihyperglycemic and antihyperinsulinemic activities by enhancing the activation of AMP-activated protein kinase (AMPK), ACC, insulin receptor, insulin-receptor substrate, and protein kinase B. AMPK activation attenuates the transcription and translation of lipogenic genes such as FAS and SCD1 and increases ACC protein expression [32]. This finding is consistent with our results. It is worth noting that the GLXBBX decoction treats hyperlipidemia induced by P407 by inhibiting the synthesis of TGs via the AMPK/SREBP1c/SCD1 signaling pathway.

Previous evidence [33] has shown that systemic LPL deficiency causes lipid accumulation in tissues (liver and skeletal muscle), which ultimately results in impaired glucose tolerance. Consistent with this finding, Li et al. [34] found that the glucose tolerance of LPL gene knockout heterozygous mice was impaired. P407-induced hyperlipidemia was accompanied by high random blood glucose levels and impaired glucose tolerance. However, GLXBBX had no effect on improving blood glucose levels. Therefore, the impaired glucose tolerance induced by P407 was not only due to the inhibition of LPL activity, but also failed to be reversed by the GLXBBX decoction.

ALT and the AST/ALT ratio have been associated with non-alcoholic fatty liver disease and insulin resistance [35]. Our results revealed that the plasma AST and AST/ALT ratio of rats with P407-induced hyperlipidemia significantly improved. Moreover, GLXBBX treatment reduced both plasma AST levels and the AST/ALT ratio.

In the present study, it is important to mention that the model rats exhibited a strong stress response during the course of the experiment. Specifically, after all the rats were sacrificed, a strong stress response was observed in the organs of the model rats, with two rats showing severe bloody edema of the kidney. In contrast, both the control rats and GLXBBX-treated rats were in good condition. Unfortunately, we were unable to examine this phenomenon in depth.

## Conclusion

In summary, our experimental results showed that GLXBBX treatment significantly regulated hyperlipidemia-like symptoms and ameliorated dysfunctional hepatic lipid metabolism in rats with P407-induced hyperlipidemia. The latent mechanisms of action may involve suppressing the mRNA expression of *SCD1* and fortifying LPL activity to reduce TG levels. The present experimental findings confirm the therapeutic effect of GLXBBX and provide a scientific rationale for prescribing this herbal formula for lipid-lowering treatment. Further work must be conducted to explore the mechanism by which GLXBBX reduces LPL activity.

## Data Availability

All supporting data are included in the main article. There are no links or online datasets.

## Abbreviations

ACC: acetyl-CoA carboxylase
ALT: alanine aminotransferase
AMPK: AMP-activated protein kinase
AST: aspartate aminotransferase
FAS: fatty acid synthase
GLXBBX: Gualou Xiebai Banxia
GPIHBP1: glycosylphosphatidyl inositol-anchored high-density lipoprotein-binding protein 1
H&E: Hematoxylin and Eosin
i.p.: intraperitoneal
LDL: low-density lipoprotein
LPL: lipoprotein lipase
ox-LDL: oxidation low lipoprotein
P407: Poloxamer 407
PPAR: peroxisome proliferator-activated receptor
qRT-PCR: quantitative reverse transcription polymerase chain reaction
RT-qPCR: real-time quantitative polymerase chain reaction
SCD1: stearoyl-CoA desaturase
SREBP1c: sterol regulatory element binding protein
TC: total cholesterol
TG: triglyceride
TLR4: Toll-like receptor 4
